# Dietary *Lycium barbarum* Polysaccharides Support Retinal Redox Homeostasis Through Gut Microbiota–Associated Metabolic Modulation

**DOI:** 10.1002/fsn3.72215

**Published:** 2026-08-17

**Authors:** Yijing Yang, Jun Peng, Danyang Li, Pai Zhou, Ying Wang, Jian Xu, Hanyu Tan, Ying Deng, Qinghua Peng

**Affiliations:** ^1^ Hunan University of Chinese Medicine Changsha China; ^2^ Hunan Provincial Key Laboratory for the Prevention and Treatment of Ophthalmology and Otolaryngology Diseases With Chinese Medicine, Hunan University of Chinese Medicine Changsha China; ^3^ Furong Laboratory Changsha China; ^4^ Changsha Centre for Innovation in Traditional Chinese Medicine Techniques for the Prevention and Treatment of Retinal Diseases and Visual Function Protection Changsha China; ^5^ Oriental Hospital of Tongji University Shanghai China

**Keywords:** functional food, gut microbiota, *Lycium barbarum* polysaccharides, metabolic regulation, retinal homeostasis

## Abstract

Dietary polysaccharides are important bioactive components of functional foods and play a key role in modulating host metabolism through interactions with the gut microbiota. *Lycium barbarum* polysaccharides (LBP) are widely consumed as dietary supplements; however, their nutritional relevance in linking gut microbiota–associated metabolic regulation to extra‐intestinal tissue homeostasis remains incompletely understood. In this study, we investigated the effects of dietary LBP intake on gut microbiota composition, metabolic profiles, and retinal redox status using oxidatively stressed 661W photoreceptor cells and rd10 mice. In vitro, LBP exposure was associated with reduced intracellular reactive oxygen species accumulation and enhanced cellular antioxidant capacity under oxidative stress conditions. In vivo, dietary LBP intake was associated with partial preservation of retinal structural parameters, including outer nuclear layer thickness and retinal vascular features. Integrated 16S rRNA gene sequencing and untargeted metabolomics analyses demonstrated that dietary LBP intake was associated with coordinated modulation of gut microbiota composition and microbial‐derived metabolic pathways, particularly those related to pantothenate and coenzyme A biosynthesis, aromatic amino acid metabolism, and aminoacyl‐tRNA biosynthesis. These metabolic alterations were reflected in retinal metabolic profiles and accompanied by improved redox balance. Overall, this study highlights the nutritional significance of LBP as a functional polysaccharide and supports the concept that dietary modulation of gut microbiota–associated metabolism may contribute to maintaining retinal metabolic homeostasis. These findings provide a nutritional and food science perspective on the potential role of dietary polysaccharides in supporting tissue function beyond the gastrointestinal tract.

## Graphical abstract

Summary schematic illustrating the proposed association among dietary LBP intake, gut microbiota remodeling, fecal metabolic pathway modulation, retinal metabolite changes, and retinal antioxidant signaling. The model summarizes associations observed in the present study and does not imply direct causal proof.

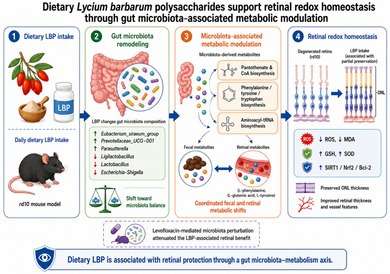

**Abbreviations**
ASVs: amplicon sequence variants
Bcl‐2: B‐cell lymphoma‐2
ELISA: enzyme‐linked immunosorbent assay
F/B: *Firmicutes*/*Bacteroidetes*
GCL: Ganglion cell layer
H&E: hematoxylin and eosin
HPLC: high‐performance liquid chromatography
IDA: information‐dependent acquisition
INL: inner nuclear layer
IPL: inner plexiform layer
IS: inner segment of photoreceptors
KEGG: Kyoto Encyclopedia of Genes and Genomes
LBP:
*Lycium barbarum* polysaccharides
Nrf2: nuclear factor erythroid‐2‐related factor 2
OCT: optical coherence tomography
ONL: outer nuclear layer
OPL: outer plexiform layer
OPLS‐DA: orthogonal partial least squares‐discriminant analysis
OS: outer segment of photoreceptors
PCA: principal component analysis
PCoA: principal coordinates Analysis
PCR: polymerase chain reaction
RP: retinitis pigmentosa
RPE: retinal pigment epithelium
SIRT1: silent information regulator factor 2‐related enzyme 1
UHPLC: ultra high‐performance liquid chromatography
UPGMA: unweighted pair group method with arithmetic mean

## 1 Introduction

Dietary polysaccharides are important bioactive constituents of functional foods and are increasingly recognized for their roles in regulating host metabolism and redox balance through interactions with the gut microbiota. As complex carbohydrates that escape digestion in the upper gastrointestinal tract, these polysaccharides serve as key substrates for microbial fermentation, thereby influencing microbial community structure and the production of bioactive metabolites relevant to systemic metabolic homeostasis (Cremonesi et al. 2022; Yang et al. 2018). *Lycium barbarum* polysaccharides (LBP), derived from goji berries, are widely consumed as dietary supplements and functional food ingredients, with reported antioxidant and metabolic regulatory properties (Ou et al. 2021; Yang, Wang, Deng, Liu, et al. 2023; Yang, Wang, Deng, Lu, et al. 2023). However, despite growing interest in LBP, their nutritional relevance in modulating gut microbiota–associated metabolism and its implications for extra‐intestinal tissue function remain insufficiently characterized (Yang, Wang, Deng, Liu, et al. 2023; Cai et al. 2017). Given the high metabolic demand and sensitivity to oxidative stress of retinal tissue, investigating nutrition‐related metabolic regulation in this context may provide insights into how dietary polysaccharides contribute to maintaining tissue redox homeostasis. Accordingly, this study examines the associations between dietary LBP intake, gut microbiota composition, metabolic profiles, and retinal redox status from a food science and nutrition perspective.

Retinitis pigmentosa (RP) is a progressive inherited retinal degenerative disorder characterized by irreversible photoreceptor loss and visual impairment, in which oxidative stress and metabolic imbalance are recognized as central contributors to disease progression (Dias et al. 2018; Léveillard and Sahel 2017). As one of the most metabolically active tissues in the body, the retina is particularly vulnerable to disturbances in redox balance and energy metabolism, making metabolic dysregulation a critical determinant of retinal functional integrity (Liu et al. 2022; Verbakel et al. 2018). Although current therapeutic strategies such as gene therapy and cell‐based approaches have shown promise, their clinical applicability remains limited (Dias et al. 2018), underscoring the importance of complementary strategies that support retinal homeostasis at the systemic level.

The gut microbiota plays a central role in mediating the nutritional functions of dietary polysaccharides by transforming complex carbohydrates into a diverse range of microbial‐derived metabolites that influence host metabolism (Parker et al. 2022). These metabolites, including short‐chain fatty acids, amino acid derivatives, and cofactor‐related intermediates, act as key signaling and metabolic molecules that link dietary intake to systemic physiological processes (Fu et al. 2023; Agus et al. 2021; Zhou et al. 2023). Increasing evidence from nutrition and food science research indicates that modulation of gut microbiota composition through dietary components can affect metabolic homeostasis in tissues beyond the gastrointestinal tract (Kutsyr et al. 2021). In this context, metabolic pathways related to energy production, redox balance, and amino acid utilization are particularly sensitive to changes in microbial activity and substrate availability. However, the extent to which specific dietary polysaccharides shape gut microbiota–associated metabolic pathways and how these changes translate into functional outcomes in metabolically active tissues remain incompletely understood. Addressing this knowledge gap is essential for clarifying the nutritional significance of functional polysaccharides and for guiding their rational application in food and nutrition research.

From a functional food perspective, this study was designed to evaluate LBP as a dietary bioactive component, rather than a pharmacological intervention, with emphasis on its associations with gut microbiota composition, microbial‐related metabolic regulation, and retinal redox homeostasis. By integrating cellular assays, a genetic mouse model of retinal degeneration, and multi‐omics analyses, we aimed to characterize how dietary LBP intake is linked to systemic metabolic pathways relevant to retinal functional integrity.

## 2 Materials and Methods

### 2.1 Reagents and Chemicals

*Lycium barbarum* polysaccharide powder with a manufacturer‐reported total polysaccharide content of 90% was obtained from Nanjing Duly Sciences Co. Ltd. (catalogue no. C0218, China). The reported purity refers to the total polysaccharide content of the commercial starting material and does not indicate a fixed monosaccharide composition. Before biological evaluation, the material was further fractionated by ion‐exchange chromatography and characterized by high‐performance anion‐exchange chromatography with pulsed amperometric detection (HPAEC‐PAD); Cell Counting Kit‐8 (CCK‐8, C0037), CM‐H2DCFDA (S0035S), Enzyme‐linked immunosorbent assay (ELISA) Lipid Peroxidation malondialdehyde (MDA) kit (S0131S), ELISA glutathione (GSH) kit (S0053), ELISA superoxide dismutase (SOD) kit (S0087), and Tunel cell apoptosis detection kit (C1086) all from Beyotime Biotechnology; Annexin V‐APC Apoptosis Detection Kit (ZY‐4102‐2, Shanghai Zeye Biotechnology Co. Ltd); Hematoxylin and Eosin (H&E) Staining Kit (C02‐04004, Beijing Biosynthesis Biotechnology Co. Ltd); B‐cell lymphoma‐2 (Bcl‐2) antibody (AF6139), Nuclear factor erythroid‐2‐related factor 2 (Nrf2) antibody (AF0639), and Silent information regulator factor 2‐related enzyme 1 (SIRT1) antibody (DF6033) were from affinity.

### 2.2 Evaluation of the Potential Activity of LBP In Vitro

#### 2.2.1 Purification of LBP and Cytotoxicity Assay‌

LBP powder (5 g) was dissolved in 200 mL of pure water and centrifuged at 10,000 *g*. The supernatant was subjected to ion‐exchange chromatography with sequential elution using water and NaCl gradients (0.1M, 0.2M, 0.3M). Total sugar content in the eluates was measured using the sulfuric acid‐phenol method and diluted to 160 g/L. For monosaccharide composition analysis, approximately 5 mg of the purified LBP preparation was hydrolyzed with 1 mL of 2 M trifluoroacetic acid in a sealed tube at 121°C for 2 h. The hydrolysate was dried under a nitrogen stream, washed two to three times with methanol to remove residual trifluoroacetic acid, redissolved in deionized water, and filtered through a 0.22‐μm membrane before analysis. Monosaccharides were separated on a Dionex CarboPac PA20 anion‐exchange column (3 × 150 mm) using an ICS 5000+ system equipped with a pulsed amperometric detector. The flow rate was 0.5 mL/min, and the injection volume was 5 μL. Monosaccharides were identified by comparison of their retention times with those of authentic standards and quantified using compound‐specific external calibration curves. The 13 standards included fucose, rhamnose, arabinose, galactose, glucose, xylose, mannose, fructose, ribose, galacturonic acid, guluronic acid, glucuronic acid, and mannuronic acid. All calibration curves showed acceptable linearity, with coefficients of determination greater than 0.99.

The 661W photoreceptor cell line was cultured in DMEM/F12 medium supplemented with penicillin–streptomycin at 37°C with 5% CO_2_. To determine the safe and effective concentration of LBP for in vitro experiments: (1) Cytotoxicity screening: 661W cells were treated with LBP at 10, 20, 40, 80, and 160 mg/L for 24 h, and cell viability was detected by CCK‐8 assay. Results showed that 10–40 mg/L LBP had no cytotoxicity, while 80–160 mg/L LBP reduced cell viability (*p* < 0.01). (2) Oxidative stress model validation: A 200 μmol/L hydrogen peroxide (H_2_O_2_)‐induced oxidative stress model was established (selected via pre‐experiment for inducing ~50% cell viability reduction). Cells were treated with 20 mg/L (low dose) or 40 mg/L (high dose) LBP, and antioxidant/antiapoptotic activities were verified by reactive oxygen species (ROS) detection, ELISA, and flow cytometry.

#### 2.2.2 Antioxidant Activity Evaluation

Oxidative stress‐induced 661W cells were treated with 20 mg/L (low dose) or 40 mg/L (high dose) purified LBP for 24 h. Intracellular ROS levels were measured using CM‐H_2_DCFDA staining (1 mmol/L, at 37°C for 30 min), followed by flow cytometry. Antioxidant capacity was quantified with ELISA kits.

#### 2.2.3 Antiapoptotic Activity Assessment

Cells treated with LBP were stained with Annexin V‐APC and propidium iodide (PI) following the apoptosis detection kit protocols, then analyzed through flow cytometry. In parallel, samples were subjected to TUNEL staining: cells were fixed with 4% paraformaldehyde, permeabilized using Triton X‐100, incubated with TUNEL reaction mix at 37°C for 1 h, and fluorescence intensity was measured using ImageJ software.

### 2.3 Animal Studies‌

Rd10 mice carrying the *Pde6b* mutation, a genetic model of retinitis pigmentosa, were obtained from Jackson Laboratory (USA), whereas wild‐type C57BL/6 mice were obtained from Hunan Slake Jingda Laboratory Animal Co. Ltd. (China). All animal procedures were reviewed and approved by the Animal Ethics Committee of Hunan University of Chinese Medicine (Approval No. LL202311030013). Mice were maintained under standard laboratory conditions at 22°C with free access to food and water.

The in vivo LBP regimens were selected based on the non‐cytotoxic and biologically responsive concentration range identified in 661W cells, together with a short‐term tolerance assessment in rd10 mice. In the tolerance assessment, rd10 mice received LBP at 20 or 40 mg/mL by daily gavage for 7 days, and no obvious body‐weight loss, diarrhea, behavioral abnormality, or gross organ injury was observed.

For the formal animal experiment, postnatal day 42 mice were assigned to five groups: wild‐type control mice receiving saline, untreated rd10 model mice receiving saline, rd10 mice receiving low‐dose LBP, rd10 mice receiving high‐dose LBP, and rd10 mice receiving levofloxacin plus high‐dose LBP. Each mouse received 0.2 mL of the assigned gavage solution once daily for 3 weeks. Based on an average body weight of approximately 20 g, the low‐dose LBP regimen of 20 mg/mL corresponded to 4 mg LBP per mouse per day, or approximately 200 mg/kg body weight/day. The high‐dose LBP regimen of 40 mg/mL corresponded to 8 mg LBP per mouse per day, or approximately 400 mg/kg body weight/day. The levofloxacin plus LBP co‐treatment group received levofloxacin together with the same high‐dose LBP regimen.

The in vitro concentration‐screening experiment was used to identify a non‐cytotoxic and biologically responsive concentration range of LBP, rather than to perform a direct pharmacological conversion to an in vivo dose. Because LBP is a high‐molecular‐weight dietary polysaccharide with limited systemic absorption and is expected to act mainly within the intestinal lumen and through gut microbiota‐associated metabolism, conventional allometric scaling based on systemic drug exposure was not applied. The selected in vivo regimens should therefore be interpreted as exploratory dietary gavage doses supported by cellular screening and short‐term animal tolerance.

Levofloxacin was included as an exploratory co‐treatment intended to interfere with microbiota‐associated responses during high‐dose LBP administration. However, fecal samples from this group were not retained for microbiome sequencing. Therefore, the extent and pattern of microbiota alteration induced by levofloxacin were not directly determined, and this group should not be interpreted as a validated microbiota‐depletion model.

### 2.4 Retinal Analysis

Retinal vasculature was imaged using Phoenix Micron IV, with vessel diameters measured at six random sites using Vessel Caliber software. Optical coherence tomography (OCT) scans assessed retinal thickness via ImageJ. After euthanasia, eyes were fixed (Powerful Biology B0006) and stained (Beyotime kit) for histological analysis of outer nuclear layer (ONL) thickness and morphology using ImageJ. Efficacy screening compared control, model, and optimal treatment groups.

### 2.5 16SrDNA Sequencing and Analysis

All five animal groups were included in retinal structural assessment. The 16S rRNA gene sequencing analysis was conducted using a focused three‐group subset comprising wild‐type C57BL/6 control mice, untreated rd10 model mice, and rd10 mice receiving high‐dose LBP. The high‐dose LBP group was selected because it exhibited the most pronounced retinal structural preservation in the phenotypic assessment. The low‐dose LBP and levofloxacin plus LBP groups were not included in the sequencing analysis. Fecal samples from the levofloxacin co‐treatment group were not retained after completion of the original experiment; consequently, retrospective microbiome sequencing of this group was not possible. The sequencing results should therefore be interpreted as an exploratory comparison of control, model, and effective high‐dose LBP conditions rather than as a complete dose–response or antibiotic‐perturbation analysis.

Fecal microbial DNA was extracted from collected fecal samples. The target 16S rRNA gene region was amplified using specific primers, and PCR products were purified using magnetic beads. DNA concentration was measured using the Qubit system, fragment size was assessed using a Bioanalyzer, and library concentration was evaluated using Illumina‐compatible quantification kits. Paired‐end sequencing was performed on the Illumina NovaSeq PE250 platform. Low‐quality sequences were filtered using fqtrim, and chimeric sequences were removed using Vsearch. Non‐redundant amplicon sequence variants (ASVs) were generated using DADA2. After sequence rarefaction, alpha and beta diversity analyses were performed. Taxonomic annotation was conducted using the SILVA database (v138), and microbial composition and diversity results were visualized using R software.

### 2.6 Metabolites Detection and Analysis

Twenty‐five milligrams of stool metabolites were separated using a Thermo Fisher Vanquish UHPLC system with a Waters BEH Amide column (2.1 × 50 mm, 1.7 μm). The Orbitrap Exploris 120 HRMS operated at resolutions of 60,000 (full scan) and 15,000 (MS/MS) with ±3.8/−3.4 kV spray voltage, 50/15 Arb gas flows, and a 320°C capillary temperature. Fragmentation used stepped SNCE (20/30/40 eV). Metabolite identification was performed using BiotreeDB v3.0, with statistical analysis in R v3.5.2. Untargeted fecal metabolomics was performed using the same three‐group subset as the 16S rRNA gene sequencing analysis, namely the wild‐type control group, the untreated rd10 model group, and the high‐dose LBP‐treated rd10 group. The low‐dose LBP and levofloxacin plus LBP groups were not included in untargeted metabolomics profiling.

### 2.7 LBP Affects Mechanisms Associated With Changed Genus and Metabolites in rd10 Mice

The differential abundance of bacterial genera and metabolites was selected to analyze the correlation. We retrieved RP‐related genes from the GeneCards database (https://www.genecards.org/) and analyzed their expression in RP versus normal groups. Venny chart analysis was used to identify the target genes associated with differential metabolites in the drug group and the differential genes for RP diseases. We further validated the metabolites changed in retina of mice and the target genes through Western blot and immunofluorescence assays.

### 2.8 Statistical Analysis

Statistical analyses were performed using GraphPad Prism 9 and R software where appropriate. Data are presented as mean ± standard error of the mean (SEM). Normality was assessed using the Shapiro–Wilk test, and homogeneity of variance was assessed using Levene's test or Brown–Forsythe test, as appropriate. For normally distributed data with homogeneous variance, comparisons among multiple groups were performed using one‐way analysis of variance followed by Bonferroni or Tukey post hoc correction. For data that did not meet parametric assumptions, non‐parametric tests were used. Independent sample comparisons were performed using Student's *t*‐test or a non‐parametric alternative according to data distribution. A two‐sided *p* value < 0.05 was considered statistically significant. Sample sizes are indicated in the corresponding figure legends.

## 3 Results

### 3.1 Characterization of LBP and Cellular Antioxidant Responses Under Oxidative Stress Conditions

To characterize the compositional features of LBP and establish appropriate experimental concentrations for subsequent analyses, purified LBP was first subjected to chemical characterization and concentration‐dependent cellular evaluation.

**FIGURE 1:**
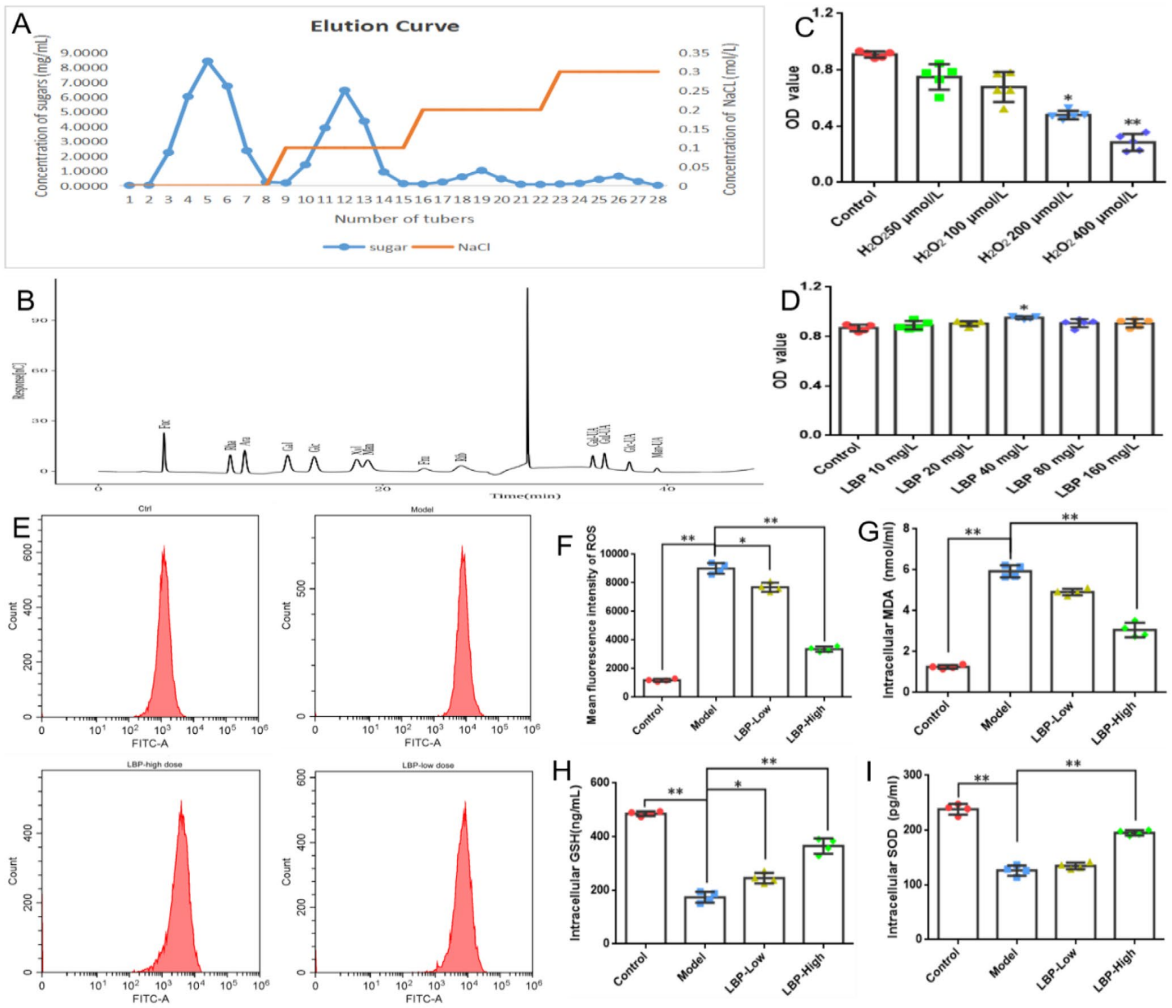
Characterization of LBP and its effects on oxidative stress in 661W cells. (A) Ion‐exchange chromatographic elution profile. (B) HPAEC‐PAD monosaccharide profile. (C) Cell viability following H_2_O_2_ exposure. (D) Cell viability following LBP exposure. (E, F) Representative ROS flow‐cytometry histograms and quantification. (G–I) MDA, GSH, and SOD levels. Data are presented as mean ± SEM. *p < 0.05; **p < 0.01.

Purified LBP exhibited two distinct carbohydrate‐associated absorption peaks, with maximal absorbance observed at 490 nm, indicating a high polysaccharide content (Figure 1A). Monosaccharide composition analysis revealed that LBP consisted predominantly of glucose, together with galactose and arabinose, as well as minor amounts of fucose, rhamnose, xylose, mannose, fructose, ribose, and uronic acids, including galacturonic, glucuronic, mannuronic, and guluronic acids (Figure 1B). Glucose was the predominant monosaccharide, accounting for 78.3 mol% of the total quantified monosaccharides, followed by galactose (8.5 mol%) and arabinose (7.2 mol%). The remaining monosaccharides comprised fucose (0.8 mol%), rhamnose (0.7 mol%), xylose (0.4 mol%), mannose (0.3 mol%), fructose (0.5 mol%), ribose (0.7 mol%), galacturonic acid (0.8 mol%), guluronic acid (0.8 mol%), glucuronic acid (0.5 mol%), and mannuronic acid (0.5 mol%).

To establish a reproducible oxidative stress condition, 661W photoreceptor cell viability was evaluated following exposure to increasing concentrations of hydrogen peroxide. Treatment with 200 μmol/L H_2_O_2_ resulted in an approximately 50% reduction in cell viability and was therefore selected as an oxidative challenge condition for subsequent experiments (Figure 1C).

LBP exposure at concentrations up to 40 mg/L did not exhibit cytotoxic effects and was associated with improved cell viability under oxidative stress conditions, whereas higher concentrations resulted in reduced viability (Figure 1D). Based on these observations, 20 and 40 mg/L were selected as low and high dietary‐relevant concentrations for further cellular analyses.

Under oxidative stress conditions, LBP exposure was associated with a marked reduction in intracellular reactive oxygen species (ROS) levels, as assessed by flow cytometry (Figure 1E,F). Consistently, biochemical analyses demonstrated decreased malondialdehyde (MDA) content together with increased glutathione (GSH) levels and superoxide dismutase (SOD) activity in LBP‐treated cells compared with the oxidative stress model group (Figure 1G–I). These findings indicate that LBP exposure is associated with enhanced cellular antioxidant capacity under oxidative stress conditions.

### 3.2 LBP‐Associated Modulation of Apoptosis‐Related Cellular Responses Under Oxidative Stress

To further evaluate cellular responses associated with LBP exposure under oxidative stress conditions, apoptosis‐related markers were assessed using flow cytometry and TUNEL staining.

**FIGURE 2:**
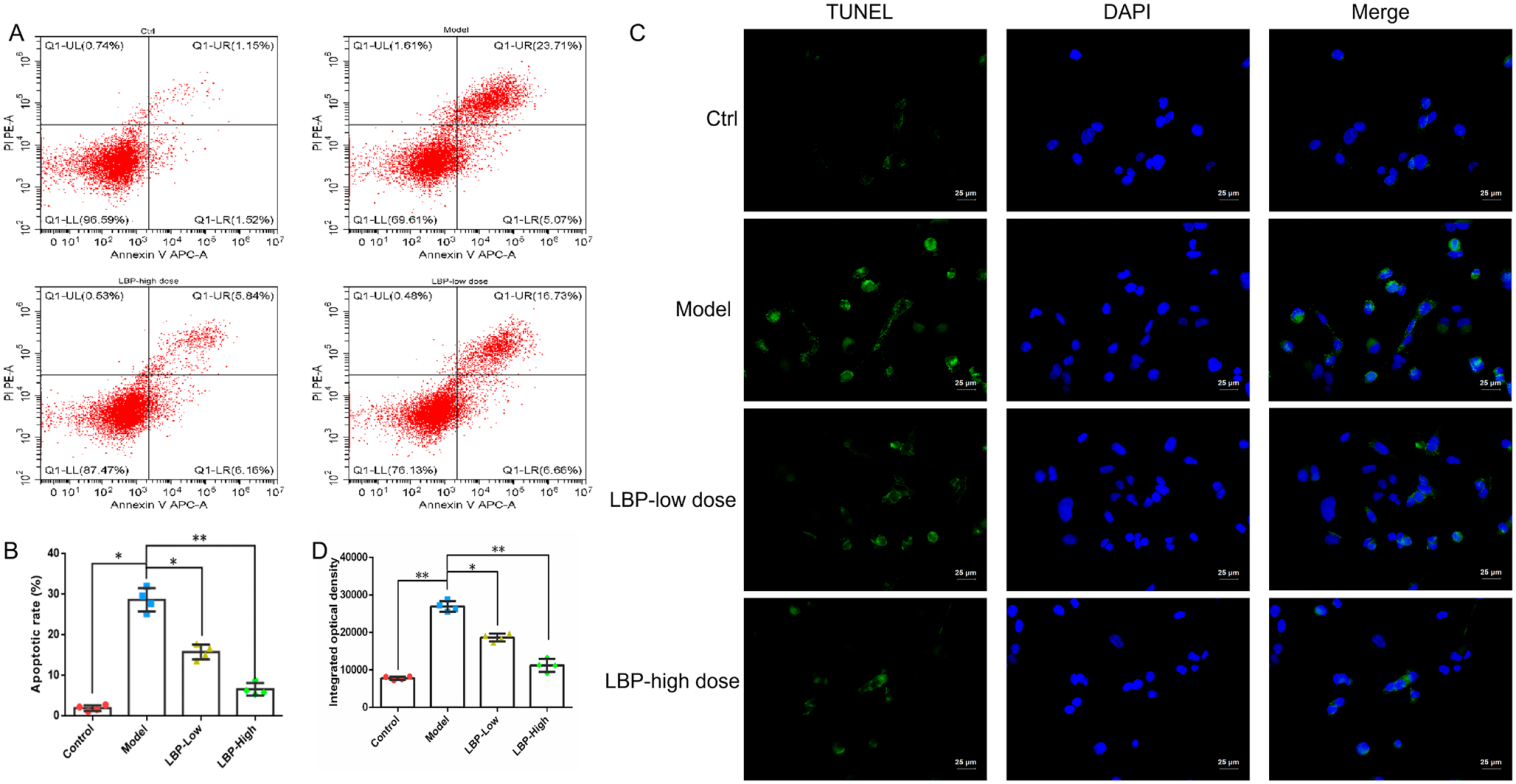
Effects of *Lycium barbarum* polysaccharides (LBP) on H_2_O_2_‐induced apoptosis in 661W cells. (A) Representative Annexin V–FITC/propidium iodide flow‐cytometry plots. (B) Quantification of apoptotic cells. (C) Representative terminal deoxynucleotidyl transferase dUTP nick‐end labeling (TUNEL) images; nuclei were counterstained with DAPI. (D) Quantification of TUNEL fluorescence intensity. Control, untreated cells; Model, H_2_O_2_‐challenged cells; LBP‐low and LBP‐high, H_2_O_2_‐challenged cells treated with 20 and 40 mg/L LBP, respectively. Scale bar, 25 μm. Data are presented as mean ± SEM. *p < 0.05 and **p < 0.01.

Flow cytometric analysis demonstrated that oxidative stress markedly increased apoptotic cell populations in 661W photoreceptor cells, whereas LBP exposure was associated with a reduction in apoptosis‐related cell populations in a concentration‐dependent manner (Figure 2A,B). Consistently, TUNEL staining revealed decreased numbers of TUNEL‐positive cells in LBP‐treated groups compared with the oxidative stress model group (Figure 2C,D).

These findings indicate that LBP exposure is associated with improved cellular survival under oxidative stress conditions, in parallel with enhanced antioxidant capacity, rather than direct modulation of a specific apoptotic signaling pathway.

### 3.3 Dietary LBP Intake Is Associated With Preservation of Retinal Structural Integrity in rd10 Mice

**FIGURE 3:**
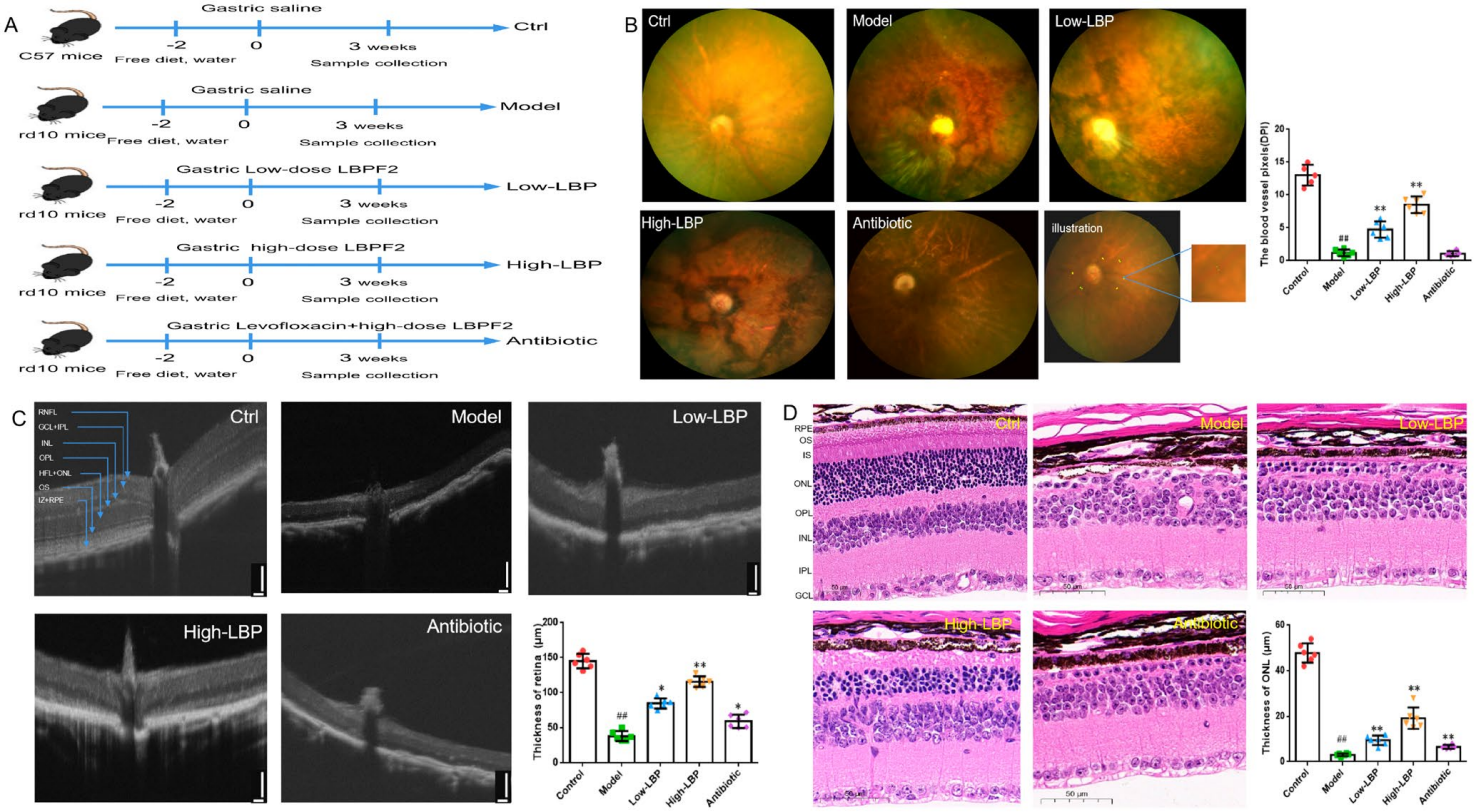
Retinal structural changes associated with dietary LBP intake in rd10 mice. (A) Experimental design. (B) Representative fundus images and quantification of retinal vessel width. (C) Representative optical coherence tomography (OCT) images and quantification of total retinal thickness. (D) Representative hematoxylin and eosin (H&E)‐stained retinal sections and quantification of outer nuclear layer (ONL) thickness. Groups comprised control, rd10 model, low‐dose LBP, high‐dose LBP, and levofloxacin plus high‐dose LBP. Scale bar, 50 μm. Data are presented as mean ± SEM. ##*p* < 0.01 versus the control group; *p < 0.05 and **p < 0.01 versus the model group.

To examine whether the cellular responses observed in vitro were reflected at the tissue level, retinal structural parameters were evaluated in rd10 mice following dietary LBP intake using fundus imaging, OCT, and histological analysis (Figure 3A).

Compared with age‐matched C57BL/6 control mice, rd10 mice exhibited characteristic features of retinal degeneration, including narrowed retinal vessels, reduced total retinal thickness, and thinning of the ONL (Figure 3B–D). In rd10 mice receiving dietary LBP, retinal vessel width and overall retinal thickness were partially preserved compared with untreated model mice, with a more pronounced association observed in the higher LBP intake group.

Histological analysis further demonstrated that dietary LBP intake was associated with partial preservation of ONL thickness, a structural indicator of photoreceptor cell density (Figure 3D). The levofloxacin plus high‐dose LBP group did not show retinal structural preservation comparable to that observed in the high‐dose LBP group. Because gut microbiota composition was not assessed in the co‐treatment group, this observation cannot determine whether the reduced retinal response was attributable to microbiota alteration or to microbiota‐independent effects of levofloxacin.

### 3.4 Dietary LBP Intake Is Associated With Alterations in Gut Microbiota Composition and Diversity

**FIGURE 4:**
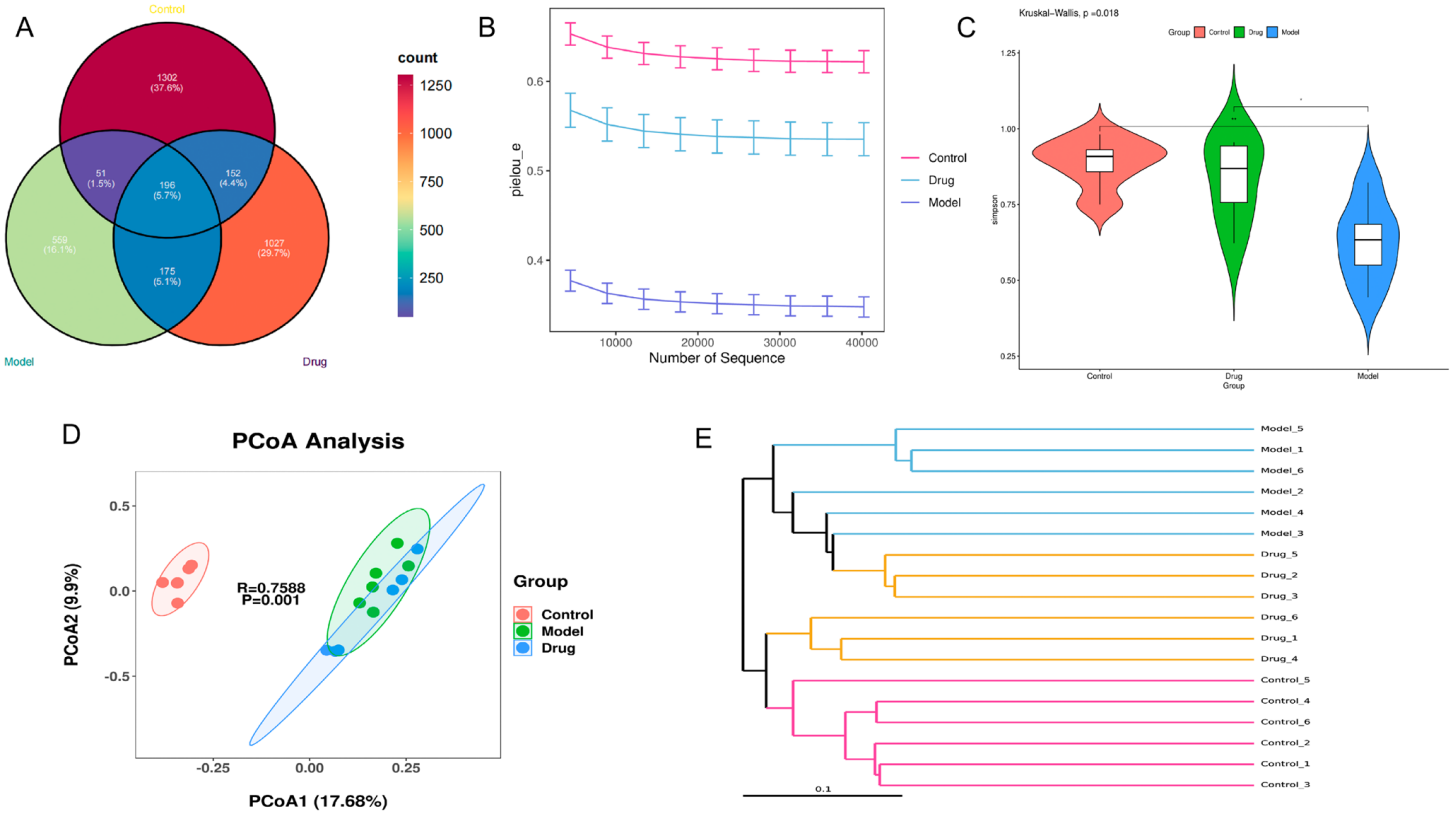
Sequencing depth and gut microbial diversity in control, rd10 model, and high‐dose LBP groups. (A) Venn diagram showing the distribution of amplicon sequence variants (ASVs). (B) Rarefaction curves. (C) Comparison of alpha diversity among the experimental groups. (D) UniFrac‐based principal coordinate analysis (PCoA). (E) Unweighted pair‐group method with arithmetic mean (UPGMA) clustering. Control, *n* = 5; model, *n* = 6; high‐dose LBP, *n* = 6.

Gut microbiota profiles associated with dietary LBP intake were characterized by 16S rRNA gene sequencing of fecal samples collected from rd10 mice. A total of 1,352,035 high‐quality sequencing reads were obtained, resulting in the identification of 3462 non‐redundant amplicon sequence variants (ASVs) across all samples (Figure 4A). Rarefaction curve analysis indicated sufficient sequencing depth to capture the majority of microbial diversity (Figure 4B).

Alpha diversity analysis revealed distinct microbial diversity distributions among experimental groups (Figure 4C), while principal coordinate analysis (PCoA) demonstrated clear separation of microbial community structures between control, model, and LBP‐treated groups (Figure 4D). Hierarchical clustering further supported distinct gut microbiota composition patterns associated with dietary LBP intake (Figure 4E).

**FIGURE 5:**
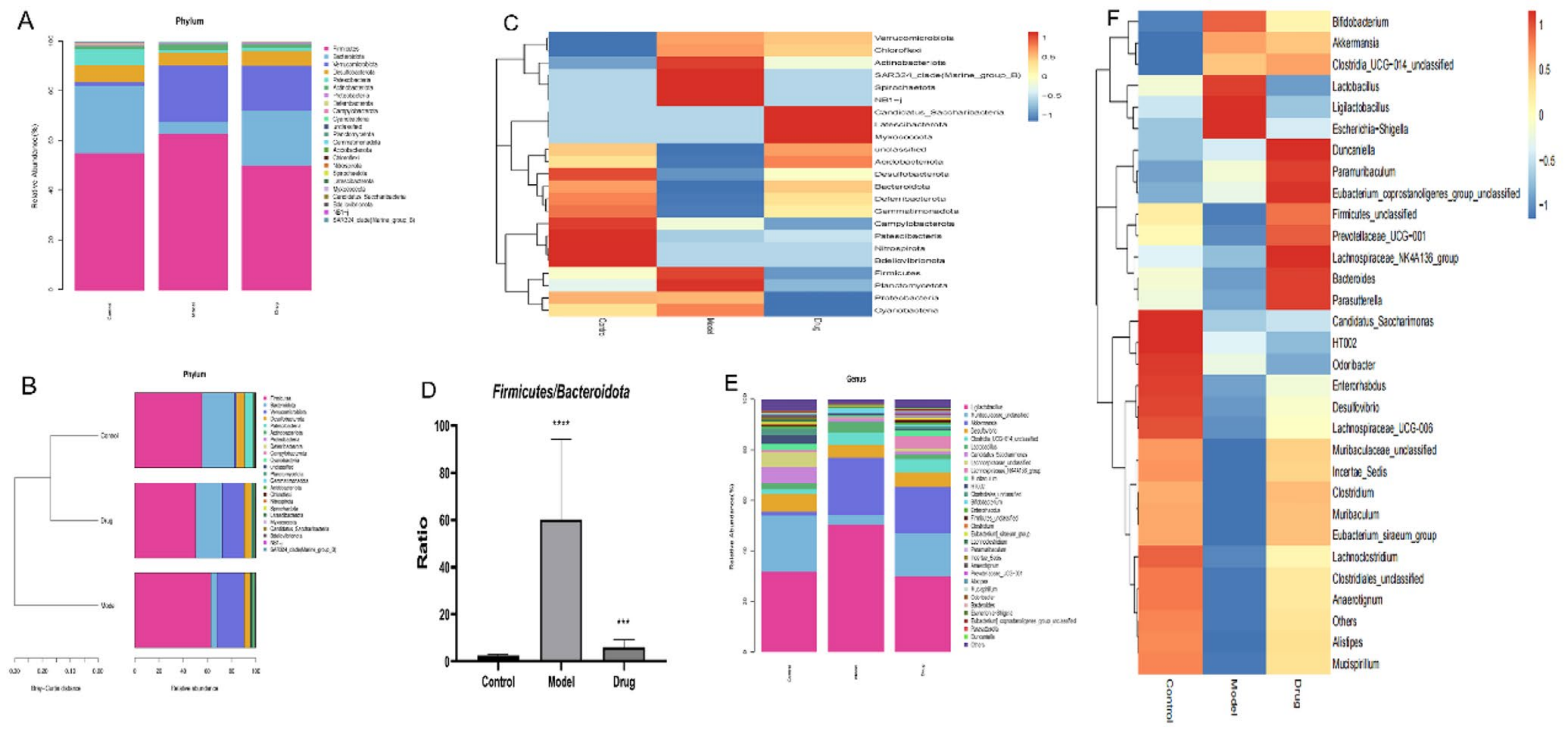
Gut microbiota composition in control, rd10 model, and high‐dose LBP‐treated mice. (A) Phylum‐level relative abundance. (B) Bray–Curtis‐based hierarchical clustering with the corresponding phylum‐level abundance profiles. (C) Heatmap of the predominant bacterial phyla. (D) Firmicutes/Bacteroidota (F/B) ratio. (E) Genus‐level relative abundance. (F) Heatmap of the predominant bacterial genera. Heatmap colors indicate row‐standardized relative abundance (red, higher; blue, lower). Control, wild‐type mice receiving saline (*n* = 5); Model, rd10 mice receiving saline (*n* = 6); LBP‐H, rd10 mice receiving high‐dose LBP (approximately 400 mg/kg/day) for 3 weeks (*n* = 6). Data in (D) are presented as mean ± SEM. ****p < 0.0001 versus Control; ***p < 0.001 versus Model. LBP, *Lycium barbarum* polysaccharides.

At the phylum level, *Firmicutes* and *Bacteroidota* represented the dominant taxa across all groups. Rd10 mice exhibited an elevated *Firmicutes*/*Bacteroidetes* (F/B) ratio compared with control mice, whereas dietary LBP intake was associated with a shift of this ratio toward control‐like levels (Figure 5A–D).

At the genus level, dietary LBP intake was associated with altered relative abundances of several microbial taxa, including increased *Eubacterium_siraeum_group*, *Prevotellaceae_UCG‐001*, and *Parasutterella*, together with decreased abundances of *Ligilactobacillus*, *Lactobacillus*, and *Escherichia–Shigella* (Figure 5E,F). Correlation analysis revealed that the relative abundances of these taxa were significantly associated with retinal structural parameters, including ONL thickness and retinal vascular width.

### 3.5 Dietary LBP Intake Is Associated With Coordinated Shifts in Fecal Metabolic Profiles

Untargeted metabolomics analysis was performed to characterize fecal metabolic profiles associated with dietary LBP intake in rd10 mice. Across all experimental groups, a total of 15,723 metabolites were detected in combined positive and negative ion modes, with quality control analyses confirming high analytical stability.

**FIGURE 6:**
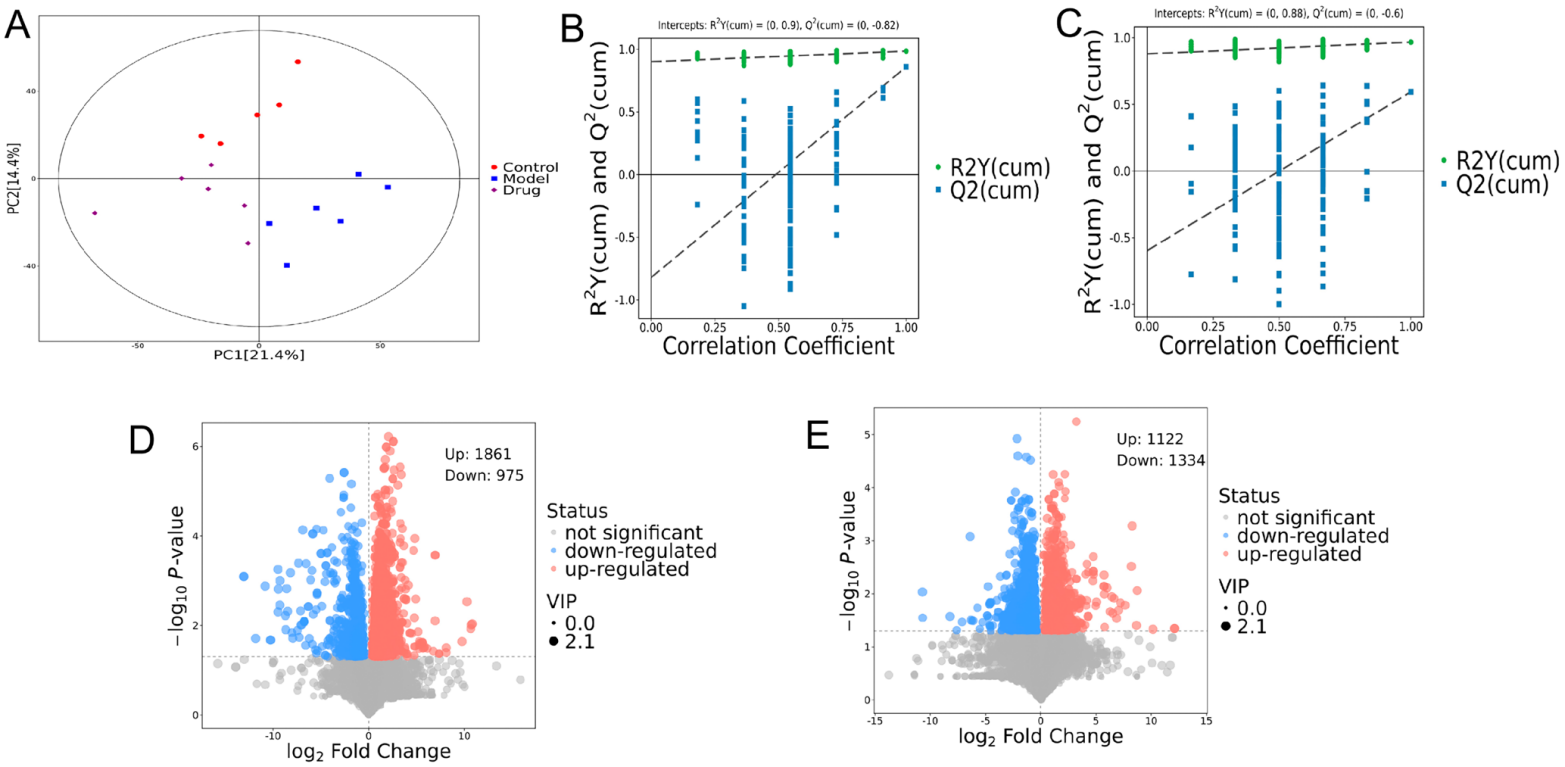
Fecal metabolomic profiles associated with high‐dose LBP intake in rd10 mice. (A) Principal component analysis (PCA) score plot. (B, C) Orthogonal partial least‐squares discriminant analysis (OPLS‐DA) permutation tests. (D) Volcano plot of differential metabolites between the control and model groups. (E) Volcano plot of differential metabolites between the model and high‐dose LBP groups. Differential metabolites were defined by a fold change (FC) > 2 or < 0.5 and *p* < 0.05. VIP, variable importance in projection.

Multivariate analyses demonstrated clear separation among control, model, and LBP‐treated groups in both principal component analysis (PCA) and orthogonal partial least squares–discriminant analysis (OPLS‐DA) score plots (Figure 6A–C). Differential metabolite analysis revealed extensive metabolic alterations in rd10 mice compared with controls, while dietary LBP intake was associated with selective shifts in metabolite abundance toward control‐like profiles (Figure 6D,E).

Notably, approximately one‐third of metabolites dysregulated in rd10 mice exhibited abundance changes in the opposite direction following dietary LBP intake, suggesting coordinated modulation of disease‐associated metabolic disturbances rather than nonspecific metabolic perturbation.

**FIGURE 7:**
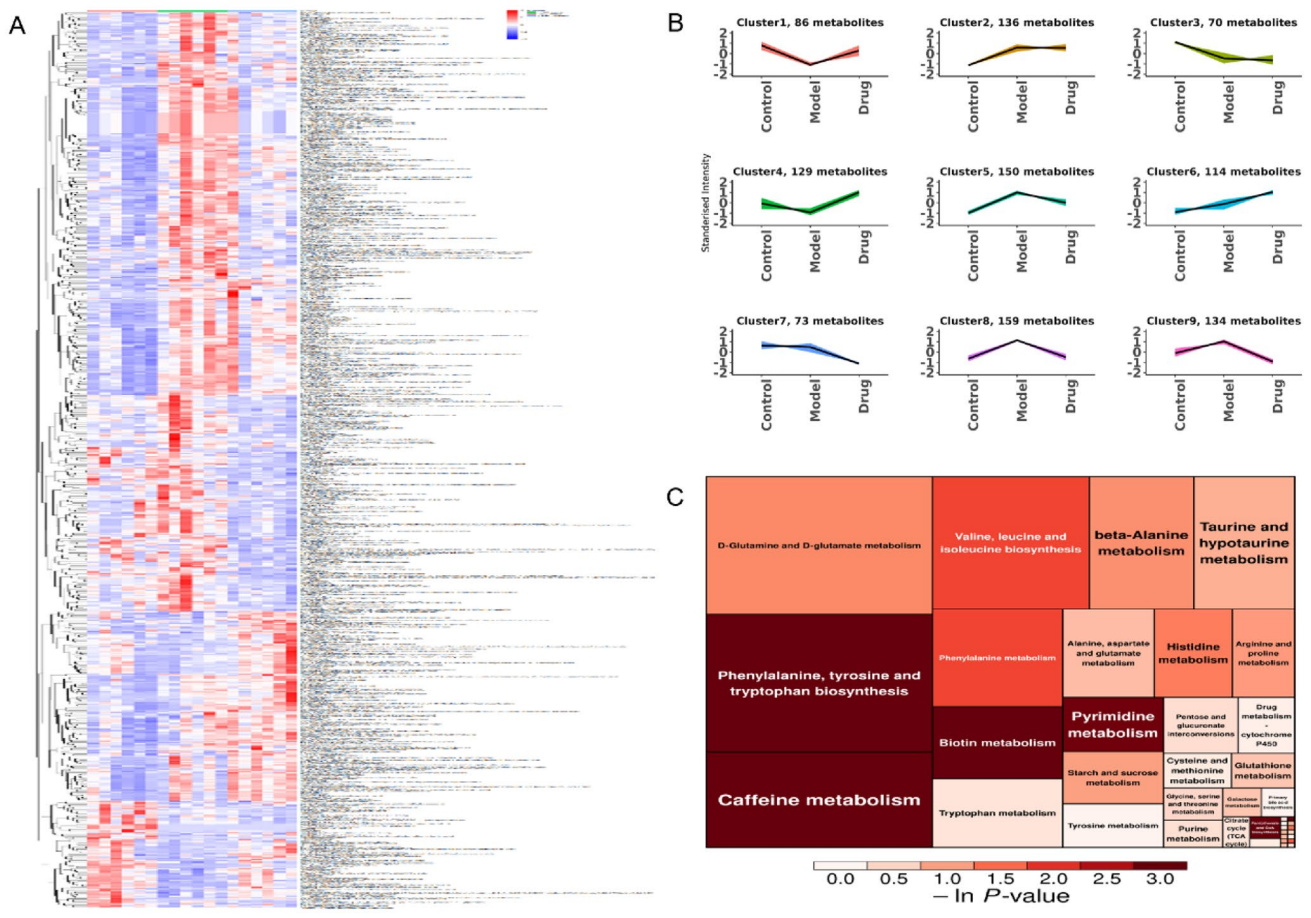
Differential fecal metabolite patterns and KEGG pathway enrichment associated with high‐dose LBP intake. (A) Hierarchically clustered heatmap of differential fecal metabolites. Rows represent metabolites and columns represent biological samples; red and blue indicate relatively higher and lower standardized abundances, respectively. (B) K‐means clustering of 1051 differential metabolites into nine clusters according to their abundance patterns across the three groups; black lines indicate the mean trend of each cluster. (C) KEGG pathway enrichment treemap. Each rectangle represents an enriched pathway, and darker red indicates greater enrichment significance based on −ln(*p* value). Differential metabolites were defined as fold change > 2 or < 0.5 and *p* < 0.05. Control, *n* = 5; Model, *n* = 6; LBP‐H, *n* = 6. KEGG, Kyoto Encyclopedia of Genes and Genomes; LBP‐H, high‐dose *Lycium barbarum* polysaccharides.

Differential fecal metabolites across all experimental groups were visualized using heatmap analysis, revealing distinct metabolite abundance patterns among control, model, and LBP‐treated rd10 mice (Figure 7A). Compared with untreated model mice, rd10 mice receiving dietary LBP exhibited metabolite profiles that shifted toward those observed in control mice, indicating an association between LBP intake and coordinated modulation of RP‐associated metabolic alterations rather than complete normalization.

K‐means clustering analysis further identified nine metabolite clusters, each displaying a characteristic abundance change pattern across experimental groups (Figure 7B). These clusters reflect coordinated metabolic responses associated with dietary LBP intake and suggest the presence of metabolite signatures linked to gut microbiota–associated metabolic regulation rather than direct therapeutic intervention.

To explore the functional relevance of these metabolite patterns, Kyoto Encyclopedia of Genes and Genomes (KEGG) pathway enrichment analysis was performed. The most significantly enriched pathways included pantothenate and coenzyme A (CoA) biosynthesis, phenylalanine/tyrosine/tryptophan biosynthesis, and aminoacyl‐tRNA biosynthesis (Figure 7C). These pathways are closely related to cellular redox balance, amino acid metabolism, and protein synthesis, supporting the notion that dietary LBP intake is associated with modulation of metabolic processes relevant to retinal metabolic homeostasis.

### 3.6 Associations Between LBP‐Related Metabolic Pathways and Retinal Antioxidant Signaling

**FIGURE 8:**
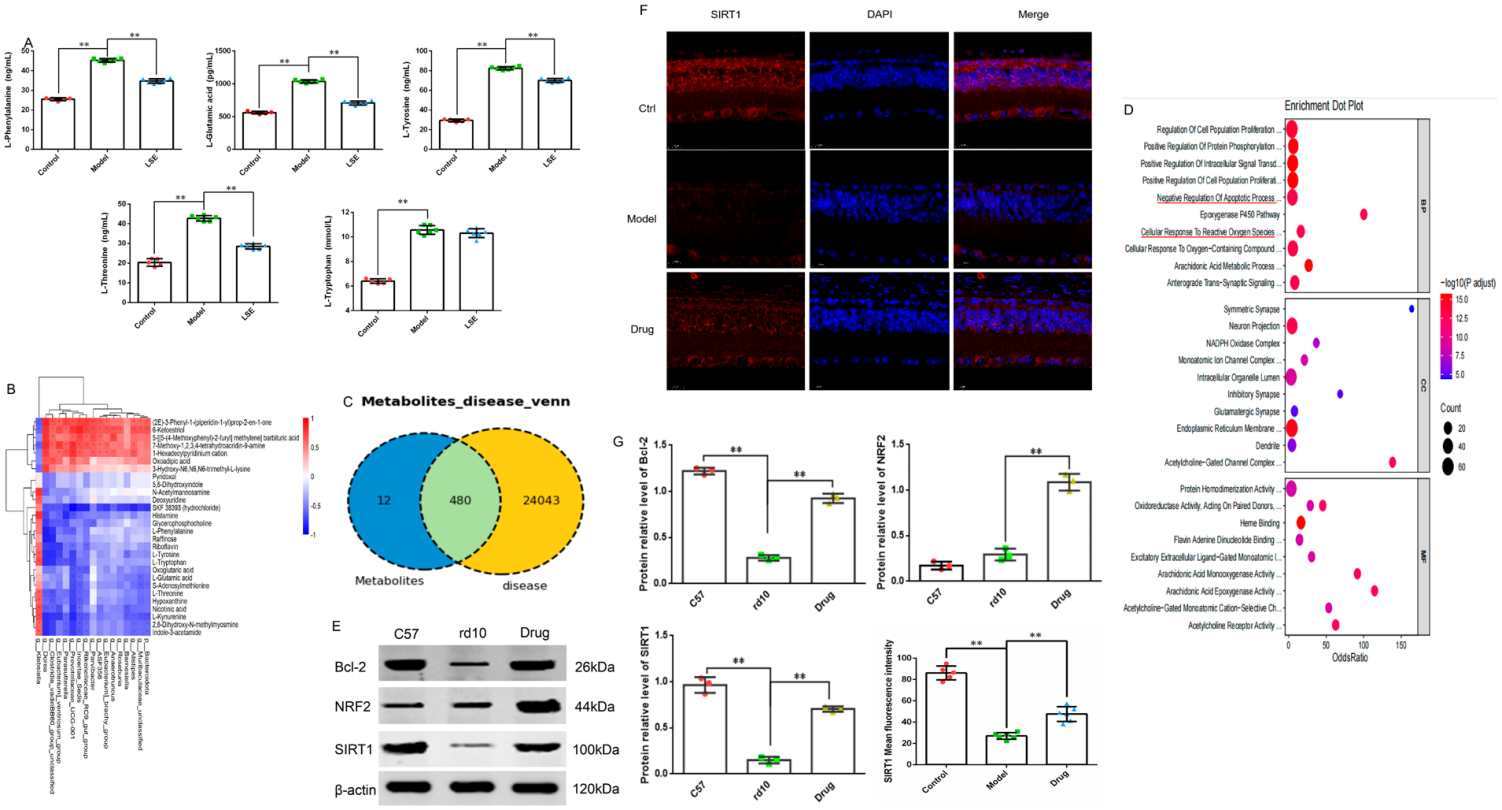
Associations of LBP‐related gut microbial and metabolic alterations with retinal redox markers. (A) Retinal levels of selected metabolites, including L‐phenylalanine, L‐glutamic acid, and L‐tyrosine. (B) Spearman correlation heatmap linking LBP‐responsive gut microbial genera with fecal and retinal metabolites. (C) Overlap between metabolite‐associated targets and retinitis pigmentosa (RP)‐related targets. (D) Gene Ontology (GO) enrichment analysis of the overlapping targets. (E) Western blot analysis of retinal sirtuin 1 (SIRT1), nuclear factor erythroid 2‐related factor 2 (Nrf2), and B‐cell lymphoma 2 (Bcl‐2) expression. (F) Representative retinal SIRT1 immunofluorescence images. (G) Densitometric and fluorescence quantification. Western blotting, *n* = 3 per group. Scale bar, 50 μm. Data are presented as mean ± SEM. **p < 0.01.

To explore potential links between dietary LBP–associated metabolic alterations and retinal molecular responses, metabolites related to key pathways were further examined in retinal tissues. Compared with untreated rd10 mice, dietary LBP intake was associated with altered retinal levels of metabolites involved in aromatic amino acid metabolism, including L‐phenylalanine, L‐glutamic acid, and L‐tyrosine (Figure 8A).

Correlation analysis revealed significant associations between LBP‐associated gut microbial genera and both fecal and retinal metabolites (Figure 8B). Integration of metabolite‐associated targets with retinitis pigmentosa–related genes identified overlapping targets enriched in biological processes related to oxidative stress regulation and cellular survival (Figure 8C,D).

Consistent with these associations, retinal expression of endogenous antioxidant and cell survival–related proteins, including SIRT1, Nrf2, and Bcl‐2, was increased in rd10 mice receiving dietary LBP, as assessed by Western blotting and immunofluorescence analyses (Figure 8E–G).

## 4 Discussion

Dietary polysaccharides are increasingly recognized as key modulators of host physiology through their capacity to shape gut microbiota composition and microbial‐derived metabolic outputs (Parker et al. 2022; Agus et al. 2021). LBP powder with reported 90% purity was further processed by ion‐exchange chromatography, and carbohydrate‐containing fractions were identified using the phenol–sulfuric acid method. Monosaccharide composition was analyzed by HPAEC‐PAD. Although these analyses characterized the major carbohydrate composition of the LBP preparation, residual non‐polysaccharide components such as trace proteins, phenolics, salts, or endotoxin were not comprehensively quantified. This limitation should be considered when interpreting the bioactivity of the preparation, and future studies should include more detailed purity and contaminant profiling. The predominance of glucose in the present preparation should be interpreted as a batch‐specific compositional feature rather than as a universal characteristic of all LBP preparations. LBP represents a structurally heterogeneous group of polysaccharides, and substantial differences in monosaccharide composition have been reported among preparations obtained using different botanical materials, extraction solvents, precipitation procedures, molecular‐weight fractions, chromatographic purification methods, hydrolysis conditions, and analytical platforms (Cai et al. 2017; Masci et al. 2018). Glucose‐rich LBP fractions have also been reported (Xia et al. 2018; Tang et al. 2015). For example, Xia et al. identified an LBP preparation in which glucose represented approximately 89.8 mol% of the quantified monosaccharides, whereas Tang et al. characterized the purified LBP3b fraction in which glucose accounted for approximately 67.8 mol%. The findings of the present study should therefore be considered most directly applicable to the chemically characterized, glucose‐rich LBP batch used here and should not be generalized to structurally distinct LBP preparations without comparative validation.

In the present study, we demonstrate that dietary *Lycium barbarum* polysaccharides, a widely consumed edible polysaccharide fraction, are associated with coordinated alterations in gut microbiota structure, fecal and retinal metabolic profiles, and retinal redox status in a genetic model of retinal degeneration. Rather than acting as a direct pharmacological agent, LBP intake appears to support retinal homeostasis indirectly through a gut microbiota–metabolism axis, providing evidence for a distal, system‐level mode of action relevant to functional foods (Kutsyr et al. 2021; Campagnoli et al. 2023).

This integrative perspective aligns with emerging views that retinal vulnerability is strongly influenced by systemic metabolic context and redox balance, rather than being determined solely by local genetic or cellular events (Dias et al. 2018; Léveillard and Sahel 2017). By positioning LBP within a dietary–microbiota–metabolism framework, the present findings extend previous work on the ocular antioxidant properties of *Lycium barbarum* –derived components (Ou et al. 2021; Yang, Wang, Deng, Liu, et al. 2023) and highlight a broader functional role for dietary polysaccharides in maintaining retinal metabolic resilience.

One important feature of dietary polysaccharides is their prebiotic‐like capacity to selectively support the growth or activity of specific gut microbial taxa (Tilg and Kaser 2011). Consistent with this concept, dietary LBP intake was associated with distinct, non‐random shifts in gut microbiota composition in rd10 mice, particularly at the genus level. Rather than inducing a generalized expansion of microbial diversity, LBP intake was linked to altered abundances of microbial taxa involved in host–microbe metabolic interactions. Such selective remodeling is characteristic of structurally complex polysaccharides and reflects differential microbial utilization rather than nonspecific prebiotic effects (Masci et al. 2018; Campagnoli et al. 2023).

Similar genus‐level specificity has been reported in studies examining food‐derived polysaccharides and phytochemicals, where dietary interventions reshape microbial communities in ways that influence downstream metabolic pathways (Zhou et al. 2023; Zhan et al. 2024). The levofloxacin plus LBP co‐treatment group did not exhibit retinal structural preservation comparable to that observed with high‐dose LBP alone. However, fecal microbiota composition was not characterized in this group, and the degree of microbiota alteration induced by levofloxacin remains unknown. Moreover, an antibiotics‐alone group was not included, and systemic or tissue‐specific effects of levofloxacin independent of the gut microbiota cannot be excluded. The co‐treatment result should therefore be interpreted as an exploratory observation rather than as evidence that an intact gut microbiota is required for the retinal effects associated with LBP intake. Future studies should include microbiome profiling of all experimental groups, antibiotics‐alone controls, broad‐spectrum antibiotic regimens, germ‐free or gnotobiotic models, and fecal microbiota transplantation to more directly evaluate microbiota dependence.

Beyond changes in microbial composition, dietary LBP intake was associated with coordinated shifts in fecal metabolic profiles, highlighting microbial‐associated metabolism as a key functional intermediary. Pathway enrichment analysis identified pantothenate and coenzyme A biosynthesis, aromatic amino acid metabolism, and aminoacyl‐tRNA biosynthesis as prominent metabolic pathways associated with dietary LBP intake. These pathways play central roles in mitochondrial energy metabolism, redox regulation, and protein synthesis—processes that are particularly critical for photoreceptor survival given the exceptionally high metabolic demand of the retina (Léveillard and Sahel 2017).

Pantothenate and CoA metabolism are essential for cellular energy homeostasis and lipid metabolism, and dysregulation of these pathways has been linked to oxidative stress and neurodegenerative vulnerability (Alessandro et al. 2012; Wang et al. 2014). Disturbances in aromatic amino acid metabolism contribute to oxidative protein modification and cellular stress responses (Ogata 2007), while impaired aminoacyl‐tRNA biosynthesis has been implicated in mitochondrial dysfunction and neurodegenerative disease processes (Yu et al. 2022). The association between dietary LBP intake and modulation of these pathways therefore suggests that microbial metabolism may act as a biochemical bridge linking dietary polysaccharides to retinal metabolic homeostasis.

The fecal metabolomic profiles indicated that dietary LBP intake was associated with a partial shift of disease‐associated metabolic alterations toward control‐like patterns, rather than complete normalization. This pattern is consistent with the concept that dietary polysaccharides act as functional food‐derived modulators that support metabolic resilience, rather than as pharmacological agents that directly correct disease‐specific molecular defects (Cremonesi et al. 2022; Zhou et al. 2023).

Pathways related to pantothenate and coenzyme A biosynthesis, aromatic amino acid metabolism, and aminoacyl‐tRNA biosynthesis may provide biochemical links between gut microbiota‐associated metabolism and retinal redox homeostasis. These pathways are relevant to mitochondrial energy metabolism, lipid metabolism, protein synthesis, and oxidative stress responses, all of which are important for photoreceptor survival in the metabolically demanding retinal environment (Tsuchiya et al. 2017; Goswami et al. 2025; Usui et al. 2009).

Under the present experimental conditions, retinal expression of endogenous antioxidants and cell survival‐related proteins, including SIRT1, Nrf2, and Bcl‐2, was increased in rd10 mice receiving dietary LBP. These molecular changes should be interpreted as indicators of improved retinal stress‐response capacity rather than as evidence of direct activation of a defined signaling pathway by LBP. This interpretation is consistent with previous studies showing that dietary antioxidants and natural products can modulate retinal oxidative stress responses in a context‐dependent manner (Ou et al. 2021; Yan et al. 2023; Moshtaghion et al. 2024). Together with the fecal and retinal metabolite results, these findings support the concept that dietary LBP may contribute to retinal metabolic homeostasis through gut microbiota‐associated metabolic regulation (Kutsyr et al. 2021; Xue et al. 2021).

The in vivo LBP doses used in this study were 200 and 400 mg/kg body weight/day, calculated from gavage concentrations of 20 and 40 mg/mL, respectively, a daily gavage volume of 0.2 mL per mouse, and an average body weight of approximately 20 g. These doses were selected as exploratory dietary gavage regimens based on cellular concentration screening and short‐term tolerance assessment in rd10 mice. Because LBP is a complex high‐molecular‐weight dietary polysaccharide that is expected to act largely through intestinal exposure and gut microbiota‐associated metabolism, direct systemic pharmacokinetic conversion from in vitro concentrations to in vivo doses was not applied. Future studies should include a broader dose range, quantitative assessment of luminal and systemic LBP‐related metabolites, and formal dose–response analyses to better define the biologically effective intake range.

The levofloxacin co‐treatment group should be interpreted as a selective microbiota‐perturbation condition rather than as a complete microbiota‐depletion model. Because an antibiotics‐alone group and omics profiling of the levofloxacin plus LBP group were not included, the present study cannot determine whether antibiotics alone affect retinal parameters or define the microbial changes induced by levofloxacin. Future studies should include antibiotics‐alone controls, broad‐spectrum antibiotic cocktails, microbiome profiling of all antibiotic‐treated groups, and fecal microbiota transplantation to more directly evaluate microbiota dependence.

Several limitations should be acknowledged. First, the present study demonstrates coordinated associations among dietary LBP intake, gut microbiota composition, microbial‐associated metabolic pathways, retinal metabolites, and retinal antioxidant signaling, but does not establish direct causality. Experimental models such as germ‐free animals, gnotobiotic colonization, fecal microbiota transplantation, and targeted metabolite supplementation or inhibition are needed to determine whether specific microbial communities or metabolites are necessary and sufficient for the retinal effects associated with LBP intake (Fritz et al. 2013).

Second, 16S rRNA gene sequencing provides primarily taxonomic information and cannot directly resolve microbial functional genes, strain‐level differences, or transcriptionally active metabolic pathways. Although untargeted metabolomics provided functional metabolic information, future studies integrating shotgun metagenomics, metatranscriptomics, and targeted metabolite validation will be necessary to identify microbial genes and pathways responsible for LBP‐associated metabolite production.

Third, rd10 mice and C57BL/6 control mice were obtained from different vendors. Vendor origin, early‐life microbial exposure, transport conditions, breeding environment, and animal facility conditions can influence baseline gut microbiota composition (Rausch et al. 2016). Therefore, the microbiome differences observed between wild‐type control mice and rd10 model mice cannot be attributed solely to genotype. In the present study, the control group should be interpreted as a reference baseline, whereas LBP‐associated omics findings should be interpreted primarily from comparisons within rd10 mice. Future studies should include vendor‐matched controls, littermate controls, co‐housing, or cross‐fostering strategies to more rigorously distinguish genotype‐dependent microbiota alterations from vendor‐related baseline differences.

Fourth, microbiome and metabolomics analyses were performed only in the control, rd10 model, and high‐dose LBP groups. Therefore, the present omics data cannot define dose‐dependent microbiota responses to LBP or directly evaluate microbiome changes induced by levofloxacin co‐treatment. Future studies should include all treatment groups in 16S rRNA gene sequencing, shotgun metagenomics, and metabolomics analyses to better establish dose–response and microbiota‐dependence relationships. Finally, the Western blot analysis was performed with a limited number of biological replicates and should be interpreted as supportive molecular evidence. Larger confirmatory studies with prospective power calculations are needed to validate these molecular findings.

A further limitation is the absence of microbiome profiling in the levofloxacin plus LBP co‐treatment group. Fecal samples from this group were not retained after the original experiment, precluding retrospective 16S rRNA gene sequencing. Consequently, the study cannot quantify the degree of levofloxacin‐induced microbiota alteration or establish a direct relationship between microbiota perturbation and attenuation of the retinal response. The co‐treatment findings should be regarded as hypothesis‐generating and require prospective validation. An additional limitation concerns the structural heterogeneity of food‐derived LBP preparations. Although the preparation used in this study was characterized by HPAEC‐PAD and showed a glucose‐rich monosaccharide profile, detailed structural features such as molecular‐weight distribution, glycosidic linkage patterns, branching degree, protein content, phenolic residues, and endotoxin content were not comprehensively determined. Consequently, the observed biological effects cannot be attributed to a single defined polysaccharide structure, and direct extrapolation to other commercially available or experimentally purified LBP preparations should be made with caution.

From a translational perspective, LBP is of interest because *Lycium barbarum* ‐derived products are widely consumed as food‐derived supplements. However, the present study was performed in cells and a mouse model and therefore cannot establish clinical efficacy in human retinal disease. Translation to human nutritional applications will require standardized LBP preparations, defined intake levels, long‐term safety evaluation, and controlled intervention studies that account for dietary background, baseline microbiome heterogeneity, disease stage, and retinal structural and functional endpoints. These challenges are particularly important because microbiota‐mediated responses to dietary polysaccharides may vary substantially among individuals.

In summary, dietary LBP intake was associated with coordinated modulation of gut microbiota composition, fecal and retinal metabolic profiles, and retinal antioxidant signaling in rd10 mice. These findings support the nutritional relevance of LBP as a functional polysaccharide and provide a basis for further investigation of gut microbiota‐associated metabolic regulation in retinal homeostasis.

## Author Contributions

**Yijing Yang:** investigation, funding acquisition, writing – original draft, writing – review and editing, formal analysis, conceptualization. **Pai Zhou:** formal analysis, writing – review and editing, investigation. **Qinghua Peng:** resources, supervision, funding acquisition, writing – review and editing, project administration. **Jian Xu:** funding acquisition, writing – review and editing, resources, validation. **Hanyu Tan:** writing – review and editing, resources, supervision. **Jun Peng:** conceptualization, investigation, writing – original draft, writing – review and editing, formal analysis. **Ying Deng:** writing – review and editing, writing – original draft, funding acquisition, supervision, formal analysis. **Ying Wang:** investigation, formal analysis, writing – review and editing. **Danyang Li:** investigation, formal analysis, writing – review and editing.

## Funding

National Natural Science Foundation of China (82274341, 82274588 and 82074196), Natural Science Foundation of Hunan Province (2024JJ5304), Key Research and Development Program of Hunan Province (2024JK2122), Scientific research project of Hunan Provincial Department of Education (23A0278, 23B0346), Open Foundation of National Key Laboratory Cultivation Base of Chinese Medicinal Powder & Innovative Medicinal Jointly Established by Province and Ministry (23PTKF001).

## Ethics Statement

The animal study was reviewed and approved by the Animal Ethics Committee of Hunan University of Chinese Medicine (Approval No. LL202311030013).

## Consent

The authors have nothing to report.

## Conflicts of Interest

The authors declare no conflicts of interest.

## Data Availability

The data that support the findings of this study are openly available in NCBI Sequence Read Archive under BioProject at https://dataview.ncbi.nlm.nih.gov/object/PRJNA1406659, reference number PRJNA1406659. Other data supporting the conclusions of this article are available from the corresponding author upon reasonable request.
